# Impact of a Shortage of First-Line Antituberculosis Medication on Tuberculosis Control — United States, 2012–2013

**Published:** 2013-05-24

**Authors:** Jon Warkentin, Jennifer Flood, Lisa True, Jennifer Kanouse, Neha Shah, Sundari R. Mase, Ann Cronin, Terence Chorba

**Affiliations:** Tennessee Dept of Health; Div of Communicable Diseases Control, Center for Infectious Diseases, California Dept of Public Health; National Tuberculosis Controllers Assn; Div of Tuberculosis Elimination, National Center for HIV, Viral Hepatitis, STD, and TB Prevention, CDC

Tuberculosis (TB) disease is treated in most cases with a regimen of several drugs taken for 6–9 months. Currently, 10 drugs are approved by the Food and Drug Administration (FDA) for treatment of TB. Of these, the four drugs that form the core for first-line treatment regimens are isoniazid (INH), rifampin, ethambutol, and pyrazinamide. In November 2012, the United States began to experience a severe interruption in the supply of INH. To assess the extent of the problem and its impact on TB control programs, a nationwide survey of programs was conducted in January 2013 by the National Tuberculosis Controllers Association (NTCA). The results indicated that the INH shortage* was interfering with patient care and could contribute to TB transmission in the United States. This report summarizes the findings of that survey, which showed that 79% of the responding health departments reported difficulties with procuring INH within the last month, with 15% reporting that they no longer had INH and 41% reporting that they would no longer have a supply within 1 month of the survey. Because of local interruptions in INH supply, responding TB programs were changing INH suppliers (69%), prioritizing patients for treatment of latent TB infection (LTBI) (72%), delaying LTBI treatment (68%), and changing to alternative LTBI treatment regimens (88%). Potential solutions for alleviating the INH shortage and averting future shortages include maintaining a national supply of first-line drugs, sharing INH among jurisdictions, working with the World Health Organization’s Global Drug Facility to obtain INH from foreign manufacturers, and strengthening reporting of shortages and impending shortages by drug suppliers to FDA.

*Mycobacterium tuberculosis* is transmitted person-to-person via the airborne route. Before the introduction of anti-TB medications, patient isolation was the principal public health intervention to minimize the risk for TB transmission. The introduction of anti-TB medications, beginning with para-aminosalicylic acid in 1944 and followed by INH in 1951, revolutionized the treatment of TB and the approach to TB control (1). Although TB continues to be a leading infectious cause of death globally (2), most patients with TB can be cured with treatment. Standard treatment worldwide for confirmed or suspected TB disease is based on the four first-line bactericidal drugs (INH, rifampin, ethambutol, and pyrazinamide), of which INH and rifampin are the most effective. Treatment of TB disease with second-line drugs can be less effective, more toxic, and more costly than treatment with first-line drugs; thus, second-line drug treatment regimens are reserved for persons with TB disease caused by INH- and rifampin-resistant strains. Additionally, INH is the recommended prophylaxis to prevent active TB disease in persons with LTBI. Alternative regimens for LTBI include rifampin and a combination of INH and rifapentine.

In 2012, three suppliers provided INH in the United States: Teva, VersaPharm, and Sandoz. A shortage of INH 300 mg tablets was first reported to CDC in November 2012; one supplier attributed the shortage, in part, to difficulty in procuring the active ingredient. The suppliers first reported an anticipated release of INH in late December 2012, but that forecast was changed to mid-January 2013. In December 2012, INH was available in 100 mg tablets, and CDC encouraged TB control programs to work with their pharmacies to obtain this formulation until the shortage of 300 mg tablets was resolved. The anticipated additional release by mid-January did not materialize, and the supply of INH 100 mg tablets also became limited.

On January 11, 2013, NTCA, an organization of state, local, and territorial public health officials and professionals, surveyed 68 jurisdictions in 50 states, 10 large cities, five territories, and three freely associated island states, using a web-based questionnaire. The questionnaire addressed issues regarding medication procurement, medication supply, and TB treatment practices related to the INH shortage.

Of the 68 surveyed jurisdictions, 42 (62%) responded. Of those responding, 38 (90%) represented state TB programs, and four (10%) represented large cities; respondents represented areas with low, medium, and high numbers of TB cases in 2011 (Figure). Of those responding to the individual questions, 33 of 42 (79%) stated they had difficulty obtaining INH within the last month, with 18 of 30 (60%) able to obtain the 100 mg INH formulation and 20 of 29 (69%) changing suppliers to obtain INH (Table). At the time of the survey, six of 39 (15%) had run out of INH, and 12 of 29 (41%) anticipated running out within 1 month. Because of the shortage, 18 of 25 (72%) programs were prioritizing only high-risk patients for LTBI treatment, 22 of 25 (88%) had changed to alternative LTBI treatment regimens (e.g., rifampin for 4 months), and 17 of 25 (68%) were delaying LTBI treatment. Because of interruptions in the INH supply, 14 of 32 (44%) programs switched to regimens that were more expensive, 37 of 37 (100%) were engaged in other activities (i.e., contacting medication distributors, issuing health alerts, modifying protocols, or answering calls), and 32 of 39 (82%) were answering calls of concern about INH supplies from the community (Table).

## Editorial Note

Interruptions in the supply of second-line anti-TB medications have been ongoing in the United States for several years (3), but since November 2012, TB control programs have experienced the first sustained generalized supply interruption of a first-line anti-TB medication. In January 2013, VersaPharm announced it would not be producing INH until 2014, leaving two manufacturers in the U.S. market. Although the two remaining manufacturers were able to begin supplying limited quantities of INH as of February 2013, the INH shortage has continued to affect TB programs. In collaboration with CDC and FDA, Teva reserved 10% of its INH supply for emergency allocation for public health programs that have been unable to access INH through their usual procurement channels. CDC and NTCA assisted Teva with developing guidance for distribution of this emergency allocation and communicated the guidance to TB programs on February 1, 2013.

The NTCA survey results show that the INH shortage has affected TB control efforts nationally. An INH shortage can directly affect patients and the community by necessitating treatment with alternative regimens that can be more expensive and, for the treatment of TB disease, more toxic. Currently a shortage also exists for two combination preparations of INH and rifampin (IsonaRif, VersaPharm; and Rifamate, Sanofi-Aventis). Increasing the use of rifampin as an alternative treatment regimen could lead to additional shortages of rifampin, the most effective drug for treating active TB.† Additionally, using an alternative preparation of INH, such as a combination INH/rifampin capsule, or using INH procured from a compounding pharmacy could be more expensive to TB programs. In many states, community health workers are unable to administer compounded drugs, requiring health departments to redirect nurses from other tasks to deliver TB therapy. If access to INH continues to be problematic, more delays or interruptions in treatment would be likely as TB programs switch to different regimens that require new protocols and additional staff training. Many programs continue to prioritize INH usage and defer treatment for many LTBI patients in accord with CDC recommendations issued on January 28, 2013, for programs with limited INH supplies (4). However, deferment of treatment for LTBI can lead to missed opportunities for TB case prevention because asymptomatic persons might be less likely to return at a later date to initiate LTBI treatment and might progress to TB disease.

What is already known on this topic?

Drug shortages, defined as situations in which the total supply of all clinically interchangeable versions of a given Food and Drug Administration–regulated drug are inadequate to meet the current or projected user demand, are a well-documented problem. Interruptions in supplies of second-line anti-tuberculosis drugs have been reported in recent years.

What is added by this report?

A nationwide survey of U.S. tuberculosis (TB) control programs in January 2013 showed that 79% of responding jurisdictions had experienced difficulty obtaining the first-line anti-tuberculosis drug, isoniazid (INH), with 15% saying they no longer had INH at the time of the survey and 41% reporting that they expected to have a shortage of INH within 1 month. The survey indicated that the INH shortage had forced TB programs to change suppliers, prioritize patients at high risk, delay treatment of persons with latent TB infection (LTBI), and change to alternative LTBI treatment regimens.

What are the implications for public health practice?

Potential solutions for improving continuity of first-line anti-TB drug supplies include the sharing of drugs in short supply among state and local TB programs, creating a drug shortage early warning system, centralized drug distribution, obtaining drugs from foreign manufacturers when drugs are unavailable in the United States, and improving the timeliness of the reporting of drug shortages by drug suppliers.

CDC, NTCA, state and local TB programs, the Treatment Action Group, and the TB Drug Shortage Working Group of the Advisory Council for the Elimination of TB are collaborating to identify short-term and long-term solutions to address the INH shortage. In addition to issuing a health advisory (4) and assisting with guidance regarding Teva’s emergency allocation, CDC, NTCA, and state and local TB programs have been implementing short-term solutions to minimize the impact of the INH shortage by using strategies such as sharing of drugs among state and local programs, using alternative formulations (e.g., substituting INH 100 mg tablets for INH 300 mg tablets), and using alternative regimens for treatment of LTBI (e.g., rifampin 600 mg daily for 4 months or INH 900 mg plus rifapentine 900 mg once weekly for 12 weeks, instead of INH 300 mg daily for 9 months). CDC reported on the shortage in December 2012 (5) and has collaborated with FDA to provide real-time updates on INH availability to TB programs through the FDA drug shortage website. CDC also has collaborated with the Southeastern National Tuberculosis Center and Treatment Action Group to conduct national meetings regarding the U.S. drug shortage. Additionally, CDC is investigating the prospect of obtaining INH from the Global Drug Facility that provides anti-TB medications to TB programs internationally. Such activities require significant investment of time and resources that could be used for other important TB control activities (6).

A 2011 presidential executive order, Reducing Prescription Drug Shortages, requires “drug manufacturers to provide adequate advance notice of manufacturing discontinuances that could lead to shortages of drugs that are life supporting or life sustaining, or that prevent debilitating disease” (7). Such advance notification of a potential INH shortage could have helped TB programs anticipate the shortage and begin making programmatic modification. Some possible long-term solutions include CDC maintaining a surveillance system to identify shortages and a U.S. distribution system for anti-TB drugs similar to the Global Drug Facility and to CDC’s Vaccines for Children Program, which supplies routinely used vaccines for eligible children and adolescents. Another possible strategy might be collaborating with FDA to determine whether anti-TB drugs in the pipeline might qualify for orphan-drug designation, which provides incentives for manufacturers to develop products for the treatment, diagnosis, or prevention of rare diseases or conditions.

The INH shortage was unexpected, has affected U.S. TB control efforts, and has lasted months longer than predicted. How the increased use of alternative regimens and the rising cost of INH driven by increased demand might affect the future supply of INH and other first-line anti-TB medications is uncertain. CDC is continuing to work on developing a sustainable solution that will maintain an uninterrupted supply of anti-TB drugs in the United States.

## Figures and Tables

**FIGURE:**
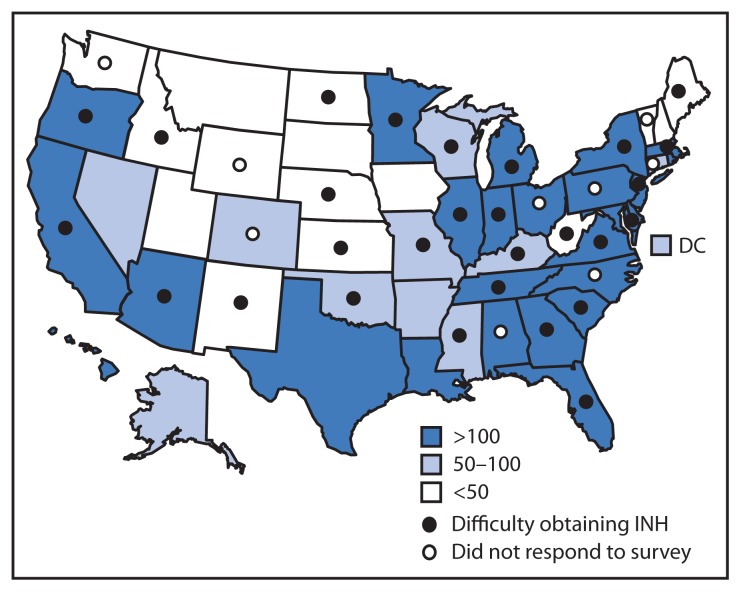
States reporting difficulty obtaining isoniazid (INH) during 2012–2013^*^ and state tuberculosis case counts in 2011 — National Tuberculosis Controllers Association survey, United States ^*^ As of January 2013.

**TABLE:** Number and percentage of jurisdictions reporting difficulty obtaining isoniazid (INH) during 2012–2013,* by effects on tuberculosis control program — National Tuberculosis Controllers Association survey, United States

Effect	No./Respondents†	(%)
**Procurement**		
Difficulty obtaining INH in last month	33/42	(79)
Able to obtain 100 mg INH tablets during 300 mg tablet shortage	18/30	(60)
Changed suppliers to procure medications	20/29	(69)
**Anticipated supply**		
No longer had supply of INH at time of survey	6/39	(15)
Would no longer have supply of INH within 1 months of survey	12/29	(41)
Would no longer have supply of INH within 1–3 months of survey	13/29	(45)
**LTBI management**		
Prioritizing certain populations for LTBI therapy	18/25	(72)
Changed to alternative LTBI regimen	22/25	(88)
Delaying treatment of LTBI	17/25	(68)
**Program resources**		
Increased cost to change regimens	14/32	(44)
Additional activities to address drug shortage§	37/37	(100)
More than one of the additional activities	31/37	(84)
Answering calls from patients, providers, nursing homes, or corrections facilities	32/39	(82)

**Abbreviation:** LTBI = latent tuberculosis infection.

* As of January 2013.

† Denominators varied because respondents were not required to answer all questions.

§ Any of the following: contacting medication distributors, issuing health alerts, modifying protocols, or answering calls.

## Footnotes

* Defined as situations in which supplies of all clinically interchangeable versions of a Food and Drug Administration (FDA)–regulated drug become inadequate to meet current or projected user demand.

† In an informal webinar survey conducted February 5, 2013, 6% of respondents reported experiencing shortages of rifampin.
